# Female genital tract melanoma: Analysis from a regional cancer institute

**DOI:** 10.4274/tjod.galenos.2020.44789

**Published:** 2020-04-06

**Authors:** Garima Pandey, Pariseema Dave, Shilpa Patel, Bijal Patel, Ruchi Arora, Chetna Parekh, Dimpy Begum

**Affiliations:** 1 Gujarat Cancer and Research Institute, Clinic of Gynae Oncology, Ahmadabad, India

**Keywords:** Genital melanoma, chemotherapy, primary surgery

## Abstract

**Objective:**

Malignant melanoma of the genital tract comprises 3% of all melanomas afflicting females. They are characterized by poor prognosis with 5-year survival of 0-25% and high incidence for distant metastasis. This study was performed to assess various clinical features, treatment options, and thre management of genital melanomas.

**Materials and Methods:**

This was a retrospective analysis where records of patients with genital melanomas between 2005 to 2018 were reviewed to obtain demographic and clinical information, including age of diagnosis, presenting symptoms, performance status, pathology reports, treatment, follow-up, and survival.

**Results:**

Between 2005 and 2018, 31 women were analyzed. The median age was 53.5 (range: 28.5-85) years. Vaginal bleeding was the most common presenting symptom (80.6%), followed by discharge (29%), mass in the vagina/perineum (19.3%), pain (16.1%), and difficulty in micturition (9.6%). The most common site of origin was the vagina (67.7%), followed by that vulva (19.3%) and cervix (12.9%). Tumor diameter was more than 3 cm in 74.2% (23/31). Out of 31 patients, only 16 opted for treatment. Four patients underwent surgery, 10 received primary chemotherapy, and two needed palliative radiotherapy for heavy bleeding. The median survival in the treatment group was 5 (range: 2.5-28) months, almost similar to patients not receiving any treatment (5 months, range: 2-11).

**Conclusion:**

Genital melanoma are rare but aggressive tumors. Diagnosis is usually made with biopsy. No effective treatment strategy is yet available. However, surgery is the preferred first- line treatment, radiotherapy and chemotherapy have been used in adjuvant settings.

**PRECIS:** A retrospective analysis was performed in patients of female genital tract melanoma.

## Introduction

Malignant melanoma of the female genital tract comprises 3% of all melanomas affecting females, and 18% of mucosal melanomas^(1)^. Melanomas arising in the female genital tract primarily occur in the vulva and vagina (95% and 3%, respectively), with the urethra or cervix being rarer causes^(1)^. The first case of primary malignant vaginal melanoma was reported in 1887, and the modern literature has noted about 500 cases globally. They are characterized by poor prognosis with an overall 5-year survival rate of 0-25%, difficult local control, and a high incidence of nodal and distant metastasis^(2)^. Being rare tumors with typically late diagnosis, unsatisfactory therapy, and a relative paucity of published data, we performed this study to assess various clinical features, available treatment options, and the management of genital melanomas.

## Material and Methods

This was a retrospective data analysis of cases of genital malignant melanoma, which was approved by the local ethics committee in our regional cancer and research institute. After institutional review board (IRB) approval, we used institutional records to identify patients who were diagnosed as having genital malignant melanomas (vulva, vagina, cervix) between 1 January 1^st^, 2005, and December 31^st^, 2018. Clinical records were reviewed to obtain demographic and clinical information, including age at the time of diagnosis, presenting symptoms, Eastern Cooperative Oncology Group (ECOG) performance status, treatment, follow-upand survival. Surgical pathology reports were reviewed to obtain gross and histopathologic tumor characterstics. All histopathologic diagnoses were confirmed using immunohistochemistry by gynecologic pathologists at our institution.

Patients were classified by primary disease site: vulval, vaginal orcervical. Computed tomography/positron emission computed tomography (CT/PET-CT) and/or ultrasonography findings were collected at baseline and during the course of treatment as a part of the initial metastatic examinations or to detect and localize recurrence during follow-up. Patients were staged using the American Joint Committee on Cancer (AJCC) 7^th^ edition guidelines for vulval melanoma. Cervical melanomas were staged as per the International Federation of Gynecology and Obstetrics (FIGO) staging of cervix cancer (2018). For vaginal melanomas, clinical staging system was used for standardization. Therapies were categorized into the following groups: surgery, chemotherapy, and radiotherapy (RT). When information on survival was not available in the medical records, an attempt was made to contact the patient and/or relatives. If such information could still not be obtained, data on the patient after their last contact were censored. Overall survival (OS) was calculated from the date of the surgical diagnosis.

## Results

### Patient characterstics

Between 2005 and 2018, 31 women presented to our regional cancer institute with a diagnosis of genital tract malignant melanoma. Patient characteristics are summarized in Table 1. The median age was 53.5 (range: 28.5-85) years. Vaginal bleeding was the most common presenting symptom (80.6%), followed by discharge per vaginum (29%), mass in the vagina/perineum (19.3%), pain (16.1%), and difficulty in micturition (9.6%). In our study, the most frequent site of origin was the vagina (67.7%), followed by the vulva (19.3%) and cervix (12.9%). The tumor diameter was more than 3 cm in 74.2% (23/31) and less than 3 cm in 25.8% (8/31). Thirteen (41.9%) patients in study group had lymph node metastasis and seven (29%) had distant metastasis.

### Treatment

Out of the total 31 patients, 16 patients opted for treatment (Table 2). Four patients underwent surgery followed by adjuvant chemotherapy (CT) or RT. Primary CT was given in 10 patients and two patients received palliative RT for heavy bleeding. Fifteen patients refused treatment (Table 3). Of these, 8 (53.4%) were above age of 65 years. Tumor size was >3 cm in 12 patients (80%) and the majority were diagnosed as being in higher stages (>2b, 10/15, 66.6%). OS was less than 6 months in 11 cases (73.3%).

### Characteristics of patients (n=4) who underwent surgery

**Figure 1:**
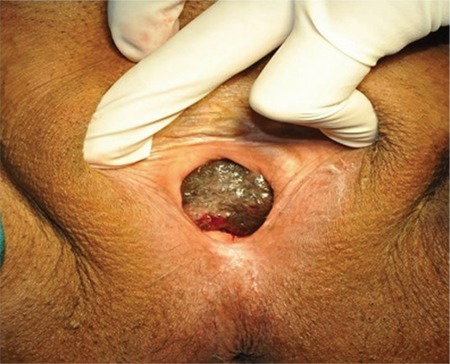
Melanoma in lower 1/3 of vagina

Among patients with surgery (n=4), one had wide local excision (WLE) with partial urethral resection (2 cm) for melanoma in the lower one-third of the vagina (Figure 1), others had radical vulvectomy with bilateral inguinofemoral lymphadenectomy for vulval melanoma, radical hysterectomy with partial vaginectomy (5 cm) for melanoma in the upper one-third of the posterior vagina, and radical hysterectomy with bilateral pelvic lymphadenectomy for cervical melanoma, respectively (Table 4). Three (75%) patients were aged less than 60 years. The median tumor size was 3.75 (range, 3-4) cm with a median depth of tumor invasion of 11 (8-15) mm. Both cases of vaginal melanoma were stage 1b, and cervical and vulval melanoma were found in stage 3c postoperatively with right internal iliac lymph node and left inguinal lymph node metastasis, respectively. The median free surgical margins were more than 17.5 (range, 15-20) mm.

As an adjuvant treatment, patients with lower one-third vaginal melanoma received external beam RT (EBRT, 50 Gy/25#), cervival melanoma received dacarbazine (400mg, 3 cycles) with tamoxifen, and vulval melanoma received temozolomide (200 mg/m^2^, days 1 to 5) plus cisplatin (25 mg/m^2^, days 1 to 3, three weekly for six cycles) with EBRT (50 GY/25#). THe patient with upper one-third vaginal melanoma had no adjuvant treatment but received temozolamide (100 mg, 5 cycles) during recurrence (rectum). She was alive at the time of the last follow-up (28 months). Overall, the median time of recurrence was 9.5 (range, 7-13) months in these four patients.

### Characteristics of patients who underwent primary chemotherapy

Among the patients who received primary chemotherapy (n=10), the majority were aged >65 years (7/10, 70%) with tumor size more than 3 cm (9/10, 90%). All of them were in advanced stages (>2b) with poor performance status in most patients (ECOG 2.3-7/10, 70%). The OS was less than 6 months in 40% of cases (Table 5).

## Discussion

Vulvar melanomas account for less than 1% of all melanomas, but they represent 10% of all malignant tumors involving the vulva^(3)^. Although vulvar melanomas arise on the hairy skin of the vulva, because of its sun-shielded location and continuity with vaginal mucosa, it has been mostly described along with mucosal melanomas. Vulvar melanomas, arising from the outer, non-glabrous hair-bearing portion of the labia majora, may share common risk factors with cutaneous melanoma. Chronic inflammatory disease, viral infections, chemical irritants, and genetic factors have also been implicated as risk factors^(4)^. However, the exact pathogenesis of vulvovaginalor cervicalmelanomas is relatively unknown. It is suggected that they arise from aberrantly located melanocytes in the vaginal epithelium found in 3% of healthy women, and in the basal portion of the vaginal epidermis and cervical melanocytic cells as embryologic remnants of neural crest cells^(5)^. Diagnosis is determined by gynecologic examination, histologic results, and immunohistochemical staining.

Although vulvar melanomas are reported to be more frequent compared with vaginal/vulvovaginal melanomas, our study reported vaginal melanomas in 67.7% of patients, vulval in 19.3%, and cervical in 12.9% of patients. In the literature, vaginal melanomas have been primarily found in the anterior wall (38%) of the vagina, mostly in the lower one-third (34%)^(6)^. Our study reported vaginal melanoma most commonly in upper half of the posterior vagina (57.1%), followed by the lower half of the anterior vagina (19%), upper half of the anterior vagina (14%), and lateral vagina (9.5%).

Genital melanomas mostly occur in older women, with a median age of 68 years in the vulva, and 60 years in the vagina and cervix^(7,8)^. The median age of patients in our study was less, 53.5 (range, 28.5-85) years. The most common presenting symptoms reported in literature are vaginal bleeding and discharge, presence of mass lesion, and less commonly, pain, pruritus or micturition difficulties. Our study also found vaginal bleeding to be the most common symptom (80.6%), followed by discharge (29%), mass lesion (19.3%), pain (16.1%), and difficulty in micturition (9.6%).

There is no universal staging system for genital melanomas. Current staging is performed as per the AJCC system for vulval melanoma, which is based on tumor thickness and the status of regional lymph nodes^(9)^. By contrast, the clinical presentation and spread pattern of malignant melanoma of the cervix is similar to that of squamous cell carcinoma of the cervix, thus the FIGO staging system has been accepted by most researchers^(8)^. In the absence of a prognostic staging system for vaginal melanomas, clinical staging system was used for standardization^(1)^.

Genital melanomas had been associated with poor prognosis, irrespective of the stage of tumor and treatment modality used. Keeping this in mind, the patients were counseled regarding disease prognosis and management options. As a result, only 16 patients opted for further treatment; four were offered surgery, 10 underwent primary chemotherapy, and two received palliative RT. Other contributing factors for refusing treatment were as follows: advanced disease stage (lesion >3 cm, 80%; stages >2b, 66%), low socioeconomic status and poor performance status (5/15, 33.3%). However, some patients may have taken the treatment outside our institute, which could not be accounted for because of the lack of a central registry of malignancies in our country.

Surgery is the main treatment option for genital tract melanoma. As expected, patients who were medically fit and had lesions amenable to surgical resection were planned for surgery in our institute. The literature on cutaneous melanoma supports a 10-mm margin for melanomas, 2 mm thick and less, and a 20-mm margin for melanomas more than 2 mm thick, which accounted for all patients in our study. Although we were able to achieve negative margins in our patients (median 17.5 mm), it required resection of the distal urethra (2 cm) in one patient due to the anatomic location of the lesion (lower one-third of the vagina).

To completely resect the primary lesion with adequate margins, the spectrum of surgery has ranged from conservative WLE to radical (vaginectomy, radical hysterectomy, radicalvulvectomy, pelvic exenteration). Without a clear improvement in survival, more conservative surgery followed by adjuvant therapy has been considered for women with genital melanoma^(1)^. Lymph node dissection is not recommended because as the rate of lymph node metastasis is low with no survival benefits^(10)^. However, in cases of clinically involved lymph nodes, lymphadenectomy can be performed. Our patient with vulval melanoma with left inguinal lymph node metastasis underwent inguinofemoral lymphadenectomy.

Attempts have been made to evaluate the benefits of adjuvant treatment after surgery. *RT* is recommended as a postoperative adjuvant therapy in patients with tumor size >3 cm or incomplete tumor resection^(11)^. Temam et al.^(12) ^found a relative risk of recurrence of 0.4 (95% CI: 0.2-0.9) in patients with mucosal melanoma who received adjuvant radiation therapy after surgery, which did not translate to improvement in OS. Frumovitz et al.^(13)^ also found that although adjuvant RT might reduce the risk of local recurrence, there was no difference in recurrence rates or survival between patients who did and did not receive adjuvant RT because the risk of distant metastasis was not reduced. Two of our patients in the surgery group received adjuvant RT due to a large tumor size, suboptimal margins, and recurrence with distant metastasis at 7.5 months.

RT is not recommended as a primay treatment but it was used in our study as a hemostatic agent in two patients for intractable bleeding per vaginum. Similiarly, the role of CT is not well established in genital melanomas, but it has been used in adjuvant settings or in advanced cases, with palliative intent..In our study, 10 patients received primary CT and four were given CT in an adjuvant setting. The patients who received primary CT in our study were older (age >65 years, 70%), advanced stage (>2b, 100%), had larger tumor size (>3 cm, 90%) with poor performance status (ECOG 2.3-70%). Multiple cytotoxic agents including dacarbazine, temozolomide and platinum compounds were used, both as single agents and in combination. The median survival was 5 (range, 2.5-18) months, almost similar to that in patients who received no treatment (5 months, range, 2-11). Postoperative adjuvant immunotherapy using interferon alpha-2b has been used to prevent relapse and has been approved by the United States Food and Drug Administration for the treatment of metastasized melanoma. Topical treatment with 5% immiquimod has been reported for preventing relapse^(14)^. However, immunotherapy could not be used at our institute due to financial constraints.

Our study has various limitations. First, almost half of study population refused treatment, irrespective of age, stage or size of lesion. It is possible that some of these may have received treatment elsewhere but could not be traced. This emphasizes the need for a central registry of malignancies in our country, but this is difficult to achieve in the forseeable future. Second, there was a high rate of loss to follow-up, so accurate information regarding recurrence or OS was unavailable. Third, the long observational study period could have added treatment heterogenicity. Although any meaningful statistical analysis could not done in any of the treatment arm, this is usually not feasible in rare diseases due to small sample sizes.

## Conclusion

Genital melanomas are rare but aggressive tumors that affect women in their 6^th^ and 7^th^ decade of life. It usually has a poor prognosis because it is often diagnosed at advanced stages with no effective treatment strategy available. Diagnosis is usually made with biopsy and immunohistochemical analysis. Primary surgery is the preferred first-line treatment. RT and chemotherapy have been used in the adjuvant setting with benefit.

## Figures and Tables

**Table 1:**
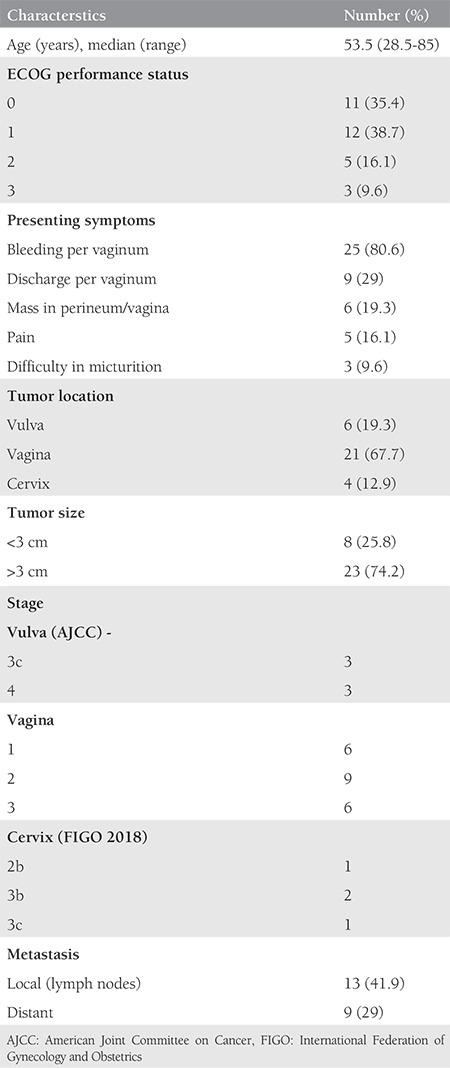
Patient characterstics (n=31)

**Table 2:**
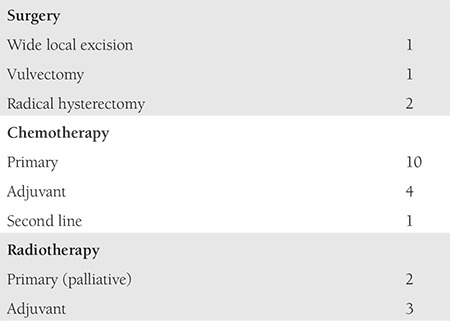
Treatment administered (n=16)

**Table 3:**
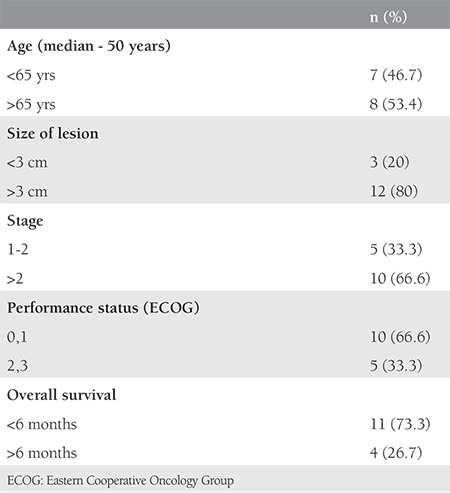
Patients who did not receive treatment (n=15)

**Table 4:**
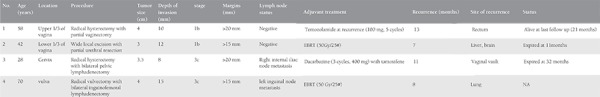
Characteristics of patients (n=4) who underwent surgery

**Table 5:**
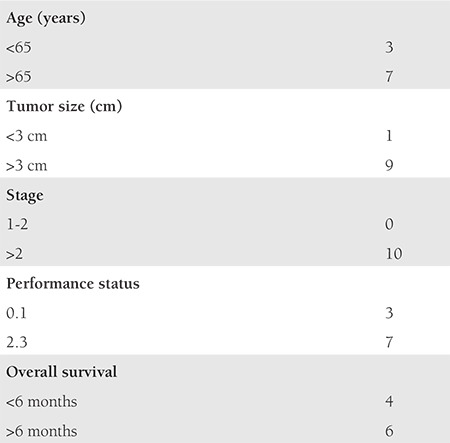
Characteristics of patients (n=10) who underwent primary chemotherapy
